# Coadaptation between Mother and Offspring: Why Not?

**DOI:** 10.1371/journal.pbio.1002085

**Published:** 2015-03-18

**Authors:** Jason B. Wolf, Michael Cowley, Andrew Ward

**Affiliations:** 1 Department of Biology & Biochemistry and Centre for Regenerative Medicine, University of Bath, Bath, United Kingdom; 2 Department of Biological Sciences and Center for Human Health and the Environment, North Carolina State University, Raleigh, North Carolina, United States of America; Institute of Science and Technology Austria (IST Austria), AUSTRIA

## Abstract

A Formal Comment has challenged the interpretation of a study into an imprinted gene, maintaining that conflict, rather than mother-offspring co-adaptation, provides a better mechanistic explanation. Here authors of the original Research Article reply.

The Kinship Theory provides a compelling explanation for the evolution of genomic imprinting at many loci. However, it need not explain the appearance of imprinting at all loci to be a valid theory, given that other processes can potentially favour imprinting [1]. Therefore, despite its predominance in the literature, we do not believe that we must necessarily consider the Kinship Theory as the default theory, even if we can construct a compatible scenario. Consequently, when hypothesizing about the evolution of imprinting of *Grb10* [2], we have suggested that the appearance of complementary effects of *Grb10* in mothers and pups is consistent with the expectation of the coadaptation theory [3]; when mothers and pups share the same expression status, pups show the “normal” phenotype, which could potentially favour maternal expression in pups. We agree that the Kinship Theory can likewise potentially accommodate evolution of *Grb10* as a maternally expressed inhibitor of early growth. Therefore, the two theories provide contradictory scenarios for *Grb10* imprinting in pups that cannot be readily differentiated using existing data.

In our discussion of imprinting at *Grb10* in mothers and pups [2], we logically focused particularly on explanations for imprinting arising as a consequence of parent–offspring interactions and hypothesized that maternal *Grb10* expression in the mother’s mammary gland could simply be a by-product of selection favouring maternal expression in pups (since the original maternal-offspring coadaptation model is indifferent as to whether the gene is imprinted in mothers [3]). In contrast, in the Formal Comment by Úbeda and Gardner (ÚAG) [4], they apply their extensions to the Kinship Theory [5,6] to hypothesize that *Grb10* imprinting in the mammary gland arises from conflict over allocare in communal nests occupied by mothers who are more related through their fathers than through their mothers. However, it is important to appreciate the scenario being assumed by ÚAG. They are assuming that mothers are able to differentially allocate provisioning to their own pups (maternal care) versus to those from other mothers (allocare) and that the two forms of provisioning necessarily trade off. Consequently, they are assuming that increased *Grb10* expression by mothers results in those mothers providing more resources to their own pups at the direct expense of their contribution to allocare. However, many studies under both lab and seminatural conditions suggest that mothers do not discriminate when providing care to their own or alien pups [e.g., 7–10], implying that care in a communal nest is nonexcludable and therefore is a “common good.” Consequently, increased *Grb10* expression is likely to modulate both maternal care and allocare simultaneously, with increased expression leading to increased total provisioning to a communal nest. Thus, the kinship model can be used to make simple and clear predictions, but the scenario assumed by ÚAG about the relationship between maternal care and allocare appear to be at odds with our understanding of the biology of communal nests. Therefore, despite conjecture to the contrary, the theory does not necessarily cleanly fit with the biology of communal care and requires further data to generate strong predictions.

Furthermore, just as the argument from ÚAG arises from their theoretical extensions of the kinship model to interactions of relatives, including those in communal nests [6], so too can the coadaptation model be extended. Using an extension of the coadaptation model, it can easily be shown that, if communally nesting females are more related through their mothers than their fathers, selection should favour maternal expression to achieve extended coadaptation through allocare (O’Brien and Wolf, unpublished manuscript). Under this scenario, imprinting causes the allocare received by pups to be more similar to that provided by their own mothers, since the relatedness asymmetry means that those mothers are more likely to share the same expressed allele. Consequently, while ÚAG suggest that, under the kinship model, mothers in communal nests should be more related through their paternally inherited allele than their maternally inherited allele, we suggest that, under the coadaptation scenario, the opposite pattern is expected. Empirical data on such relatedness asymmetries are lacking, so firm conclusions cannot be drawn.

ÚAG also raise the issue of how the traits we studied ultimately relate to fitness, which we agree is a crucial question. They focus on offspring body mass, assuming that larger pups have higher fitness. However, we dispute this argument for two reasons. Most importantly, in our study the larger pups that they assign higher fitness to are also much leaner than “normal” (wild-type) pups. Body fat gained under maternal care is presumably an important determinant of postweaning survival, and therefore we caution ascribing higher fitness to the larger pups without direct data on fitness. There is also very limited evidence that larger pups actually have higher fitness. The argument from ÚAG that larger pups have higher fitness is built on results from a single paper [11], self-described as a “preliminary study,” which simply shows that adult male (but not female) social rank is correlated with adult weight, which itself is correlated with weaning weight. While it may be true that larger adult males have higher social rank, there is no reason to believe that total natural selection (which includes all forms of selection) actually favours larger males (given that there is typically ample genetic variation for body size to evolve rapidly [12], but natural populations of mice are presumably not getting larger). Thus, while it is possible that larger pups have higher fitness, more data are clearly needed to relate body size and body composition of pups to lifetime fitness.

We are pleased that our original paper stimulated such a discussion of evolutionary processes despite our primary focus being the extraordinary pattern of expression and functions of *Grb10* [2]. We avoided overstating the evolutionary arguments given the lack of direct evidence, and it is clear from the arguments present here and in ÚAG that critical evidence explaining the evolution of imprinting at this fascinating gene is still lacking.
